# Meteorological Table

**Published:** 1811-10

**Authors:** 


					[ ssi 3
METEOROLOGICAL TABLE.
From August 26, to September -6.
D
0
Therm.
Atraos. vanatioa. v
a
62 66 56
52 68 6
53 66 6l;
56 70 621
Sll55 73 62
11*6 68 60
2157 65 5Si
' 60 62 59i303
62 68 60130J
64- 69 62
66 70 61
59 70 60
8157 70 61
9 58 70 64.
10 59 72 61
11
12
13
14
15
U
17
61 76 721G01
65 73 65
51 70 60
57 71 61
61 70 hS
61 70 58
59 69 60!
32 70 59130
19)59 70 611299
20\62 71 68 29s
62 66 60^296
60 65 60 29s
61 59 56 297
51 62 55,296
25)59 55 5229
26,50 60 49 293
18!
p uant'ty of rain from August 26 to September 2G, of an inch.
*ailir?m l^e 3ot'1 ?f August to the lfith gf September uninterrupted dry weather, the pre-
Wlnt*s E* and N. E. the hygrometer during this period indicating a very small
of a ?f raoisture in the air. On the J 6 th a few drops of riin fell, and an appearancc
?n th , n&c occurred, which however did not t ike place fully until the -23d. Lightning
23 d *"C and t^un^er on the 21st. The dry state of the atmosphere continued to the
lhe hygrometer indicated a gradual approach to humidity from the 20th.
^hiclf most remarkable meteorological fact of this interval is the appearance of a Comet,
M. pn w ,s first seen at Viviers by M. de Fianguergue on the 25th of Match, and again by
this t-nS 3t MaTSe''Hes on the 25th of April, and on the 2oth of May at Paris. From
f?tm' 'I"0 ''became invisible until the 21st of August, when it was observed in England,
1 diaS('^l ^ne w'lh the two stars in the hinder foot of Una major, its appear-
aud i^as l^en nebulous, the denser light 5 or G rriin. diameter, strongest in the cer.ire,
ing au e a star of the 4th magnitude ; the general ciiffu-ed coma thin and delicate, txtend-
nak^2 & de?ree- ^ has since become more defined, and has been distinctly v^ibie to
briHj eye fr?m the 7th of September. l^r. Olbers of Bremen states, that its greatest
I)CCe ncy wi'1 be in the month of October, and that it will continue to be visible until
*an 2q* .^^eform> the magnitude, (the tail ot this Comet is asserted to he more
0 miNk>ns of miles in length), and the effects these bodies, on their near approach
352
to the earth, may produce on its atmosphere, are objects of. grelat s6licitude.
Though the present philosophy does not admit that disastrous events,^ wars, and
disea?es, are shaken from their beards. Comets cannot be contemplated with in-
difference. A supposed possibility of their passing near the Earth's orbit may create
some alarm; and before the time ot Sir Isaac Newton, who demonstrated that
they wfue a pajrt of the Solar.System, revolving round the Sun in long ellipses,
this alarm always rose into terror. The present ^nlighfened state of science leads*
however, to a conclusion, that their path in the Solar System is defined an^ des-
tined, and that their interference with the orbits of other planers is no more pro-
bable, than that Saturn or Jupiter should wander from their spheres, and fall
those of Mars or Mercury*
PritiCts Street, Cavendish Square,
1 " 1 1 , ? ? LUJ 1 i. ,j . .. ? -

				

